# Objective Measurement of Hyperactivity Using Mobile Sensing and Machine Learning: Pilot Study

**DOI:** 10.2196/35803

**Published:** 2022-04-25

**Authors:** Oliver Lindhiem, Mayank Goel, Sam Shaaban, Kristie J Mak, Prerna Chikersal, Jamie Feldman, Jordan L Harris

**Affiliations:** 1 Department of Psychiatry School of Medicine University of Pittsburgh Pittsburgh, PA United States; 2 Human-Computer Interaction Institute School of Computer Science Carnegie Mellon University Pittsburgh, PA United States; 3 NuRelm, Inc Pittsburgh, PA United States; 4 University of Pittsburgh Medical Center Pittsburgh, PA United States

**Keywords:** assessment, machine learning, hyperactivity, attention-deficit/hyperactivity disorder, ADHD, wearables

## Abstract

**Background:**

Although hyperactivity is a core symptom of attention-deficit/hyperactivity disorder (ADHD), there are no objective measures that are widely used in clinical settings.

**Objective:**

We describe the development of a smartwatch app to measure hyperactivity in school-age children. The LemurDx prototype is a software system for smartwatches that uses wearable sensor technology and machine learning to measure hyperactivity. The goal is to differentiate children with ADHD combined presentation (a combination of inattentive and hyperactive/impulsive presentations) or predominantly hyperactive/impulsive presentation from children with typical levels of activity.

**Methods:**

In this pilot study, we recruited 30 children, aged 6 to 11 years, to wear a smartwatch with the LemurDx app for 2 days. Parents also provided activity labels for 30-minute intervals to help train the algorithm. Half of the participants had ADHD combined presentation or predominantly hyperactive/impulsive presentation (n=15), and half were in the healthy control group (n=15).

**Results:**

The results indicated high usability scores and an overall diagnostic accuracy of 0.89 (sensitivity=0.93; specificity=0.86) when the motion sensor output was paired with the activity labels.

**Conclusions:**

State-of-the-art sensors and machine learning may provide a promising avenue for the objective measurement of hyperactivity.

## Introduction

### Attention-Deficit/Hyperactivity Disorder and the Need for Objective Measurement of Hyperactivity

ADHD is the most common neurodevelopmental disorder of early childhood, affecting over 5% of American children [1]. There are 3 presentations of ADHD: (1) predominantly inattentive presentation, (2) predominantly hyperactive/impulsive presentation, and (3) combined presentation. In school-age children, ADHD predominantly hyperactive/impulsive presentation and combined presentation make up 55% of all ADHD cases [2]. Although there are objective assessment tools such as the Conners Continuous Performance Test 3rd Edition (Conners CPT 3) to measure inattention (the core symptom of the predominantly inattentive presentation of ADHD), there are no comparable objective assessment tools to measure hyperactivity (the core symptom of ADHD predominantly hyperactive/impulsive presentation). Instead, the current standard measurement for hyperactivity consists of subjective reports via questionnaires from parents and teachers, such as the Vanderbilt Assessment Scales. Reliance on subjective questionnaires to measure hyperactivity is a significant public health concern as it causes misdiagnosis including overdiagnosis and underdiagnosis [3-5]. Overdiagnosis can lead to unnecessary treatment, while underdiagnosis can lead to delayed treatment [6,7].

### Sensor Technology and Machine Learning

Advances in sensor technology and machine learning provide opportunities to develop new methods of diagnoses with enhanced objectivity and precision. Wearable technologies (eg, smartwatches) with state-of-the-art sensors are practical, cost-effective solutions for providing objective measures of hyperactivity in children. The wide array of sensors (eg, accelerometer) embedded in wearable technology offer new opportunities to develop objective and accurate measures of hyperactivity. Although actigraphy has been around for decades and extensively used in research settings, its use has largely been confined to sleep studies [8]. Actigraphy has been used in a limited number of studies on children with ADHD, typically to measure sleep duration [9-11], rather than quantify daytime levels of hyperactivity to aid in diagnosis.

### Multilevel Classification to Determine the Context of Symptoms

Context is critical in correctly diagnosing hyperactivity. For example, symptoms of ADHD include “often leaves seat in classroom or in other situations in which remaining seated is expected” and “often runs about or climbs excessively in situations in which it is inappropriate” [1]. While running and climbing in the playground do not contribute to a diagnosis of ADHD, running and climbing in a classroom does. Machine learning can be applied to sensor data to establish the context in which hyperactivity is present. Context is a combination of activity and situation. To assess context, we have developed a multilevel classification approach that first classifies the wearer’s activity, then contextualizes the level of motion, and finally evaluates activity level based on that context. The method analyzes hand motion to detect various activities; it collects the relationship between the wearer’s condition, activity, and magnitude of motion through accelerometer, time, and location data. Although it is not possible to have a class for every activity a user might perform (eg, fidgeting or other nonpurposeful motion), LemurDx classifies activities into common categories as a first layer of classification that is sufficient to condition algorithm parameters.

### This Study

We describe the development of a smartwatch app to measure hyperactivity in school-age children. The LemurDx prototype is a software system for smartwatches that uses built-in sensors and machine learning to measure hyperactivity, with the goal of differentiating children with ADHD combined presentation or predominantly hyperactive/impulsive presentation from children with typical levels of activity. In this pilot study, we used LemurDx and supervised machine learning models paired with activity data from 30 children to develop initial classification algorithms. We report on usability scores from the LemurDx prototype and accuracy results from the initial algorithms.

## Methods

### Overview

This pilot study tested the feasibility of collecting, storing, and analyzing motion, as well as contextual (ie, GPS, heart rate, Bluetooth) data, from children aged 6 to 11 years who wore an Apple smartwatch with the LemurDx app for 2 days. The data from the days when the participants with ADHD were unmedicated combined with contextual data extracted from the smartwatch sensors, as well as activity labels were included in the final analyses.

### Ethics Approval

The project was approved by the Institutional Review Board (19040006) at the University of Pittsburgh.

### Participants

Participants were recruited via a web-based research registry called Pitt + Me, through the University of Pittsburgh’s Clinical and Translational Science Institute program. The research staff subsequently contacted interested participants via phone to complete the eligibility screening. The sample consisted of 30 children aged 6 to 11 years (ADHD combined presentation or hyperactive presentation=15; non-ADHD=15) and their families. Inclusion criteria for the ADHD sample included a formal diagnosis of ADHD combined presentation or hyperactive presentation, which was confirmed using the ADHD module of the Kiddie Schedule for Affective Disorders and Schizophrenia Present and Lifetime Version (K-SADS-PL) diagnostic interview and a score of ≥10 on the hyperactivity items of the Vanderbilt Assessment Scale–Parent report (VAS-P). Exclusion criteria included serious child psychopathology requiring alternative treatment (eg, bipolar disorder, major depressive disorder, psychosis, autism spectrum disorder).

### Measures

#### VAS-P Report

VAS-P [12] is a 47-item survey that scores instances of behavior based on frequency of occurrence. Scoring is broken down into the following subtypes: inattentive, hyperactive/impulsive, or combined types. For the purposes of the study, only the 5 hyperactive subtype questions were asked in order to determine the level of the child’s hyperactive behavior. Symptoms are rated on a Likert scale from 0 to 3.

#### K-SADS-PL Diagnostic Interview

The K-SADS-PL [13] is a semistructured diagnostic interview designed to assess current and past episodes of psychopathology in children and adolescents according to Diagnostic and Statistical Manual criteria. Probes and objective criteria are provided to rate individual symptoms. For the purposes of the study, the research staff only asked about the items related to the ADHD diagnostic criteria located in the ADHD supplement.

#### Post-Study System Usability Questionnaire

The Post-Study System Usability Questionnaire (PSSUQ) [14] is a 19-item survey that assesses 3 factors: system usefulness, information quality, and interface quality. The survey uses a 7-point Likert scale in which participants indicate the degree to which they agree or disagree with each item. Lower scores indicate higher levels of agreement, while higher scores indicate lower levels of agreement.

#### Activity Labels

Parents also provided activity labels to help train the algorithm. The activities were logged at a 30-minute resolution. For each 30-minute increment of time, from 6 Am to midnight, the parent had a drop-down menu of 5 activity classifications that best summarized the child’s activities over the course of the 30-minute block. The activity categories were as follows: sleeping (eg, napping, sleeping); sitting/quiet activity (eg, watching TV, reading a book, using the internet, etc); everyday/household activity (eg, taking a walk, cleaning room, going shopping, playing an instrument, etc); exercise (eg, playing a sport, running, playing in the playground); and not wearing the watch. The activity labels created an additional level of qualification to the motion data collected. The purpose was to be able to later use the deidentified activity labels data to parallel the sensor data, to check the fidelity of the sensor data, and qualify outliers found in the sensor data.

### Procedure

After obtaining informed consent from the parent and assent from the child, the participants wore an Apple smartwatch with the LemurDx app running on it for 2 days. One arm of the study (n=15) consisted of children diagnosed with ADHD predominantly hyperactive/impulsive presentation or combined presentation, confirmed using the K-SADS-PL. The children wore the smartwatch for at least 1 day when they did not take their medication (eg, Saturday, Sunday, medication holidays), given that properly titrated stimulant medication reduces hyperactivity. The control arm (n=15) included children without an ADHD diagnosis, confirmed using the K-SADS-PL. The parents also provided activity labels via an automated remote assessment.

### Data Processing and Analyses

**Figure 1:**
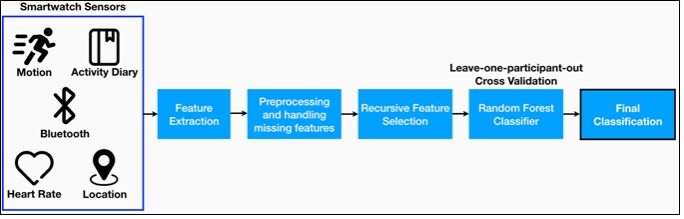
LemurDx classification pipeline.

The data processing and analysis pipeline consisted of three main steps: (1) feature extraction to calculate a set of motion and behavioral features over different time periods, (2) feature selection to identify a set of useful features and reduce the dimensionality, and (3) modeling the final set of features (Figure 1) to identify children with ADHD using a supervised machine learning approach.

### Feature Extraction

We computed 3 sets of features from the motion data collected on the watch. The first set included information about the shape of the motion curves over time and included features such as skewness and kurtosis. These features allowed us to identify the *type* of motion the children were making. The second set of features included statistical summaries of the motion data and included features such as mean, variance, median, magnitude, and hour quantiles of the observed motion. These features tend to capture both the *amount* of and the *changes* in motion. The third set of features included the cumulative motion recorded by the watch. This feature captured the total amount of motion exhibited over a time window. We calculated all 3 sets of features for 3 axes of the acceleration data and over 3 time windows, that is 1, 5, and 10 minutes. Apart from calculating these features over the course of the whole day, we also divided the features into times of the day when the children were performing specific activities as recorded in the activity labels. We used features from 3 activity classes: sitting/quiet, exercise, and everyday/household activity. Next, we handled the missing features due to missing sensor data. We occasionally missed sensor data due to technical issues with the app or watch, or compliance and human factors issues (eg, the family forgot to charge the watch). We imputed all the missing features with a value of –1.

### Feature Selection

We used the randomized logistic regression (RLR) method to select an optimal set of features before classifying the data. RLR randomly subsamples the data and calculates feature importance based on their performance in a classification task, using logistic regression [15]. This approach usually leads to a stable and reproducible set of selected features. We selected the top 20 features outputted from RLR implementation of scikit-learn.

### Modeling

We used Python’s scikit-learn library (Python Software Foundation) for the model building and for all analyses. We tried 3 types of learning algorithms: random forests, support vector machines (SVMs), and logistic regression. We chose these algorithms for their ability to generalize, capture inherent nonlinearity in the data (using specific kernels in case of SVMs), and their ability to model noisy data. Our analyses showed that random forests (with 2000 decision stumps or estimators) gave the best performance. Analyses were performed with leave-one-participant-out cross-validation to ensure that the models did not overfit. This approach builds a separate model for each participant in the validation phase and ensures that no participant’s data are shared between training and testing.

## Results

### Participant Demographics

Key demographic variables and hyperactivity scores are summarized in Table 1.

**Table 1:** Participant demographics.

Characteristics		All participants (N=30)	
		ADHD^a^ (n=15), n (%)	Non-ADHD (n=15), n (%)
Age (year), mean (SD)		9.6 (1.6)	10.1 (1.8)
**Gender, n (%)**			
	Female	6 (40)	9 (60)
	Male	8 (53)	6 (40)
	Other	1 (6.7)	0 (0)
**Race, n (%)**			
	White	11 (73.3)	14 (93.3)
	Black or African American	1 (6.7)	1 (6.7)
	More than one race	1 (6.7)	0 (0)
	Chose not to answer	2 (13.3)	0 (0)
VAS-P^b^ hyperactivity scores, mean (SD)		11.5 (2.2)	1.9 (1.7)

^a^ADHD: attention-deficit/hyperactivity disorder.

^b^VAS-P: Vanderbilt Assessment Scale*-*Parent report.

### Usability

Sensor data from the LemurDx app were successfully collected for 28 of the 30 child participants. A total of 2 participants accidentally turned off location recording in the settings of the watch. For 3 other participants, the watch failed to record heart rate. Also, 5 participants (3 in the ADHD group and 2 in the control group) had trouble recording additional sensor data. We theorized that this failure was due to a loose fit of the watch on the children’s small wrists. The overall PSSUQ usability scores were high (mean 1.81, SD 0.93) as were all the other subscale scores including usefulness (mean 1.81, SD 1.13), information quality (mean 1.75, SD 0.89), and interface quality (mean 1.92, SD 1.16). There were 3 common themes among the qualitative survey results: challenges with the app’s interface, low battery life, and participants who enjoyed using the app. Fewer than 20% of the participants had some trouble with the watch’s interface. Qualitative feedback is summarized in Table 2.

**Table 2:** Smartwatch app usability survey qualitative feedback.

Theme	Example quotes	Frequency, n (%)
Challenges with the interface	“It would be helpful if it showed something on the face of the watch to let you know that the app was running in the background.” “There was really very little we saw of the study app. Just that we turned it on, saw how long it was running for and turned it off. It's hard to say how satisfied we were with its functions.”	5 (17)
Low battery life	“We struggled with the battery running out before we were finished recording a full day’s data, despite the battery being at 100% at 7.30 AM.” “Phone ran out of battery on first day- hope that did not affect things- we can redo it if needed.”	4 (13)
Enjoyed the app	“It was fun to participate.” “Study is well organized and was easy to follow instructions.”	3 (10)

### Accuracy

The top 20 motion features extracted from motion sensor data are summarized in Table 3.

**Figure 2:**
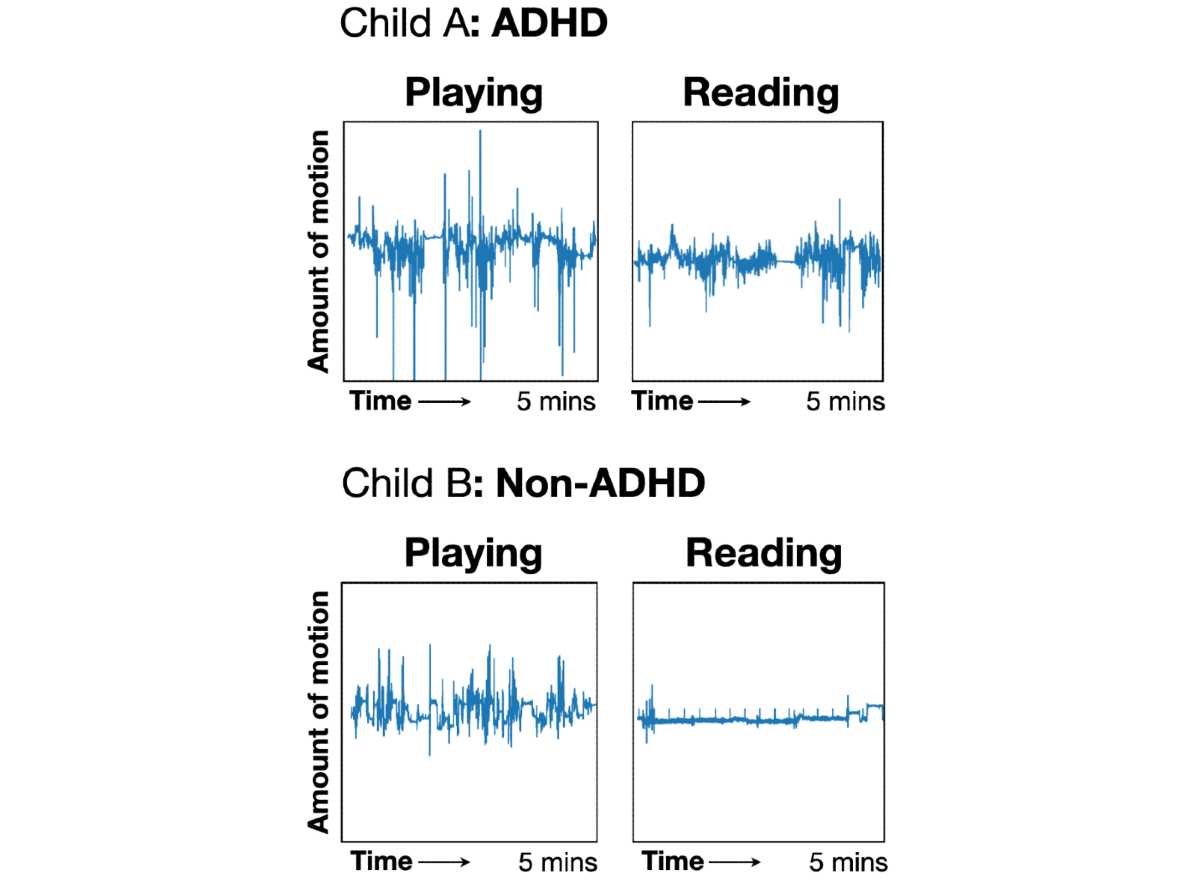
Motion spectrograms (x-axis: time; y-axis: motion) for 5-minute periods. A: The top panels are for a child with attention-deficit/hyperactivity disorder (ADHD). B: The bottom panels are for a child from the control group. In the first panel (playing), the child with ADHD moved 24.7 % more than the child in the control group. In the second panel (reading), the child with ADHD moved 41.2 % more than the child in the control group. ADHD: attention-deficit/hyperactivity disorder.

Alone, the model was no better than chance at differentiating between ADHD and non-ADHD children (accuracy=0.46; *X^2^*_1_=0.14, *P*=.70). When the motion sensor data was paired with the contextual sensors (ie, GPS, heart rate, Bluetooth), the model performance improved significantly (accuracy=0.71) and could differentiate between ADHD and non-ADHD children at better than chance level (*X^2^*_1_=5.25, *P*=.02). Model performance was best when the sensor data was paired with the activity labels (accuracy=0.89) and could reliably differentiate between ADHD and non-ADHD children (*X^2^*_1_=17.37, *P*<.001). Sensitivity, specificity, positive predictive value (PPV), and negative predictive value (NPV) for each model are summarized in Table 4. Finally, our analyses showed (Figure 2) that the magnitude of motion when the child was expected to “sit quietly” was the biggest differentiator between ADHD and non-ADHD children, consistent with a clinical profile of hyperactivity.

**Table 3:** Top 20 features extracted from motion sensors.

Number	Motion feature	Axis	Time interval
1	Cumulative variance	X-axis	10 minutes
2	Cumulative mean	X-axis	1 minute
3	Cumulative mean	X-axis	5 minutes
4	Cumulative mean	Y-axis	10 minutes
5	Cumulative variance	Z-axis	1 minute
6	Cumulative mean	All 3 axes	10 minutes
7	Cumulative variance	All 3 axes	5 minutes
8	Mean motion	X-axis	10 minutes
9	Variance	X-axis	10 minutes
10	Variance	X-axis	1 minute
11	Mean	Y-axis	10 minutes
12	Variance	Y-axis	10 minutes
13	Mean	Y-axis	1 minute
14	Variance	Y-axis	1 minute
15	Mean	Y-axis	5 minutes
16	Variance	Y-axis	5 minutes
17	Variance	Z-axis	10 minutes
18	Mean	Z-axis	1 minute
19	Variance	Z-axis	1 minute
20	Mean	Z-axis	5 minutes

**Table 4:** LemurDx accuracy, sensitivity, specificity, positive productive value (PPV), and negative productive value (NPV).

Model	Accuracy	Sensitivity	Specificity	PPV	NPV
Motion sensors plus activity labels	0.89	0.93	0.86	0.87	0.92
Motion sensors plus contextual sensors^a^	0.71	0.79	0.64	0.69	0.75
Motion sensors alone	0.46	0.50	0.43	0.47	0.46

^a^Contextual sensors included GPS, heart rate, and Bluetooth.

## Discussion

### Principal Findings

This study examined the LemurDx smartwatch app prototype among a sample of 30 children. Usability scores were high, pointing to the potential clinical utility of this approach to provide an objective measure of hyperactivity. However, qualitative feedback pointed to some issues with the interface and battery life, indicating that further development is needed in these areas. Despite these limitations, the app performed well enough to collect usable sensor data from 93% of the sample and successfully classify children with high accuracy.

As expected based on past actigraphy studies, motion data alone were a poor classifier of hyperactivity. Using motion sensors alone, model performance was no better than chance level at differentiating children with ADHD (hyperactive or combined presentations) from the ones in the healthy control group. Accuracy improved significantly when contextual information and activity labels were added to the models. These results suggest that contextual information is important when using sensor motion data to make inferences about the presence of hyperactivity.

These promising results point to the value of further research on contextualizing motion data for clinical purposes. Using the range of sensors provided in modern smartwatches could allow us to further refine the machine learning algorithms. These results would likely yield increases in accuracy based on variables specific to each child. The LemurDx app also has the potential to provide an objective measure of response to stimulant medication, thereby providing clinicians with objective data based on which medication titration decisions could be made. Overall, the data from this study support the further refinement of the LemurDx app and algorithms in order to provide an objective measure of hyperactivity to supplement the subjective parent and teacher questionnaires.

### Limitations

A limitation of this study was the absence of children with borderline levels of hyperactivity. Only children who met the specific cutoff numbers on the Vanderbilt’s hyperactivity screen were recruited. Children had to score an 8 or above to meet the ADHD condition, while a score of 5 or below was needed for the control condition. Recruiting children who scored an 8 or higher but still met the control condition would help with diversifying the data. Another limitation of this pilot study was the small sample size, as only 30 families were included in the study sample, with usable motion data from 28 children. The sample size, however, was sufficient for this preliminary pilot work. A larger sample in the future will allow for stronger indicators of context, better visualization tools for clinicians, and more precise machine learning models.

## Abbreviations

ADHD: attention-deficit/hyperactivity disorder
K-SADS-PL: Kiddie Schedule for Affective Disorders and Schizophrenia Present and Lifetime Version
PSSUQ: Post-Study System Usability Questionnaire
RLR: randomized logistic regression
SVM: support vector machine
VAS-P: Vanderbilt Assessment Scale-Parent report
